# Extracellular phosphatidylcholine functions as a noncanonical TLR4 activator to drive adipocyte lipolysis and apoptosis

**DOI:** 10.3389/fphar.2026.1783264

**Published:** 2026-04-29

**Authors:** Do Su Lim, Ki Lyong Bae, Min Kyung Pyo, Jun Hwi Ko, Jin Kyoung Choi, Tae Woo Jung, Jonghyuk Lee, A. M. Abd El-Aty, Ahmet Hacımuftuoglu, Yoon Hee Chung, Ji Hoon Jeong

**Affiliations:** 1 Department of Pharmacology, College of Medicine, Graduate School of Chung-Ang University, Seoul, Republic of Korea; 2 Department of Global Innovative Drugs, Graduate School of Chung-Ang University, Seoul, Republic of Korea; 3 Amipharm Co., Ltd., Seongnam, Republic of Korea; 4 Department of Social and Administrative Pharmacy, College of Pharmacy, Chung-Ang University, Seoul, Republic of Korea; 5 Department of Pharmacology, Faculty of Veterinary Medicine, Cairo University, Giza, Egypt; 6 Department of Medical Pharmacology, Medical Faculty, Ataturk University, Erzurum, Türkiye; 7 Department of Anatomy, College of Medicine, Chung-Ang University, Seoul, Republic of Korea

**Keywords:** apoptosis, lipolysis, nuclear factor-kappa B (NF-kB), phosphatidylcholine (PC), toll-like receptor 4 (TLR4)

## Abstract

This study investigated whether phosphatidylcholine (PC), a key active component in injection lipolysis, functions as a damage-associated molecular pattern (DAMP) via toll-like receptor 4 (TLR4) signaling to induce lipolysis and apoptosis in 3T3-L1 adipocytes. While PC is widely used for localized fat reduction, its specific molecular mechanism—beyond detergent-like necrosis—remains a subject of debate. We hypothesized that high-dose extracellular PC triggers a specific receptor-mediated inflammatory response. Our results demonstrated that PC treatment in a range of 0–25 mg/mL reduced adipocyte viability and lipid accumulation in a clear concentration-dependent manner. These catabolic effects, including increased glycerol release, were significantly and dose-dependently attenuated by pretreatment with the specific TLR4 inhibitor TAK-242 (1 or 10 nM). To definitively distinguish the mode of cell death, Annexin V/DAPI staining was performed, confirming that PC-induced cell death is driven by programmed apoptosis rather than non-specific necrosis. Mechanistically, while total protein expression of TLR4 remained unchanged, PC treatment significantly upregulated the phosphorylation of nuclear factor-kappa B (p-NF-κB) and the expression of tumor necrosis factor-alpha (TNF-α). These pro-inflammatory and apoptotic signals were effectively reversed by TLR4 inhibition, providing definitive proof that PC functions as a functional activator of the TLR4-NF-κB-TNF-α signaling axis. In conclusion, these findings demonstrate that extracellular PC acts as a noncanonical TLR4 agonist, driving adipocyte-specific lipolysis and apoptosis. This study provides a comprehensive molecular rationale for PC-based fat reduction and suggests that the modulation of TLR4 signaling could optimize the efficacy and safety of lipolytic therapies in clinical practice.

## Introduction

The global epidemic of obesity represents a major public health challenge characterized by the excessive and pathological expansion of adipose tissue. Adipose tissue is no longer viewed merely as an inert energy storage depot but is now reorganized as a dynamic endocrine organ capable of secreting a vast array of bioactive factors, collectively known as adipokines (Kershaw and Flier, 2004). This pathological accumulation of visceral and subcutaneous fat is intricately linked to a state of chronic low-grade inflammation, often termed “meta-inflammation,” which plays a pivotal role in the development of metabolic dysregulation, insulin resistance, and cardiovascular diseases (Hotamisligil, 2017). In the specific context of aesthetic medicine, excessive localized adiposity—such as in the submental region or abdomen—poses a significant psychological and social burden, driving the increasing demand for minimally invasive reduction strategies. Among these nonsurgical interventions, injection lipolysis (commonly known as mesotherapy) utilizing formulations of PC and sodium deoxycholate (DC) has emerged as a popular and effective alternative to surgical liposuction.

Despite its widespread clinical use, the precise pharmacological mechanism of injection lipolysis remains a subject of intense scientific debate. The pharmacological action of DC, a bile salt, is well established; it functions as an ionic detergent that causes nonspecific adipocyte necrosis through cell membrane lysis (Schuller-Petrovic et al., 2008; Rotunda et al., 2004). In contrast, the specific role of PCs has been historically controversial. PC was initially considered a passive vehicle or emulsifier, necessary solely for solubilizing the hydrophobic bile salt DC in an aqueous solution. However, the accumulation of clinical and experimental evidence challenges this passive view. Comparative studies of PC-containing formulations and DC alone suggest that compared with detergent-mediated lysis, PC alone has active biological effects on adipose tissue remodeling (Bechara et al., 2007). Recent *in vitro* and *in vivo* studies have demonstrated that PC-based formulations selectively induce lipolysis and apoptosis in mature adipocytes while relatively sparing nonadipose lineages such as fibroblasts and muscle cells (Jung et al., 2019; Kim et al., 2017; Li et al., 2011). This cell-type specificity, which cannot be attributed to simple chemical necrosis, strongly implies the involvement of precise, receptor-mediated signaling pathways that specifically target adipocyte biology.

We hypothesize that this mechanism is driven by the recognition of extracellular lipids by the innate immune system via TLR4. TLR4 is a transmembrane pattern recognition receptor (PRR) whose role in host defense is primarily to recognize lipopolysaccharide (LPS) from the outer membrane of gram-negative bacteria (Akira and Takeda, 2004). However, its role extends beyond pathogen recognition; TLR4 serves as a broad-spectrum sensor that detects endogenous “danger signals” or DAMPs released during tissue stress, injury, or cell death (Gong et al., 2020). These DAMPs include saturated fatty acids, heat shock proteins, high-mobility group box 1 (HMGB1), and extracellular matrix fragments. Upon activation by these ligands, TLR4 initiates a signaling cascade involving the adaptor proteins MyD88 and TRIF, ultimately leading to the translocation of NF-κB and the production of proinflammatory cytokines such as TNF-α (Kawai and Akira, 2010). TNF-α is a potent autocrine and paracrine regulator of lipid metabolism; it promotes lipolysis by downregulating perilipin (the protective coating of lipid droplets) and activating hormone-sensitive lipase (HSL) while simultaneously driving adipocytes toward programmed cell death (apoptosis) (Sethi and Hotamisligil, 1999; Ryden et al., 2002).

Although PC is a ubiquitous and essential building block of healthy eukaryotic cell membranes, its exposure in the extracellular space at high pharmacological concentrations—as seen in injection lipolysis—is an aberrant event that challenges tissue homeostasis. Structural analyses revealed that the acyl chains of saturated phospholipids can dock into the hydrophobic pocket of myeloid differentiation factor 2 (MD-2), the essential coreceptor for TLR4, thereby mimicking the biological activity of Lipid A, the toxic moiety of LPS (Maeshima and Fernandez, 2013; Park et al., 2009). Thus, we propose that high-dose extracellular PC or its metabolite acts as a pharmacological DAMP. By functioning as activators of TLR4, PCs trigger a sterile inflammatory response that culminates in programmed adipocyte death and lipid breakdown, distinct from the necrotic pathway induced by detergents (Palsson-McDermott and O'Neill, 2013).

In this study, we investigated the physiological relevance and molecular mechanism of PC as a nonclassical TLR4 activator in differentiated 3T3-L1 adipocytes, a well-characterized model of white adipose tissue. By utilizing the specific TLR4 inhibitor TAK-242 (resatorvid), which binds selectively to the intracellular domain of TLR4 to block signal transduction, we aimed to determine whether the lipolytic and apoptotic effects of PC are dependent on TLR4 signaling. Furthermore, we sought to elucidate downstream molecular events, focusing on the expression of inflammatory cytokines and apoptotic markers, to provide a comprehensive molecular rationale for the use of PC in targeted fat reduction therapies.

## Materials and methods

### Cell culture and differentiation

Murine 3T3-L1 fibroblasts were obtained from the American Type Culture Collection (ATCC; Manassas, VA, United States). Cells were cultured in Dulbecco’s modified Eagle’s medium (DMEM; Gibco) supplemented with 10% bovine calf serum (BCS; Gibco), penicillin (100 U/mL), and streptomycin (100 μg/mL) in a humidified atmosphere of 5% CO_2_ at 37 °C. To induce adipogenesis, cells were grown to postconfluence (Day 0) and then stimulated with a standard differentiation cocktail consisting of DMEM supplemented with 10% fetal bovine serum (FBS; Gibco), 1.0 μM dexamethasone (Sigma‒Aldrich), 0.5 mM 3-isobutyl-1-methylxanthine (IBMX; Sigma‒Aldrich), and 10 μg/mL insulin (Sigma‒Aldrich). After 48 h of induction (Day 2), the medium was replaced with DMEM containing 10% FBS and 10 μg/mL insulin alone to support lipid droplet formation. After another 48 h (Day 4), the cells were maintained in DMEM supplemented with 10% FBS, and the medium was changed every 2 days. After full differentiation was achieved (day 8), the cells were used for experiments, which were confirmed by Oil Red O (ORO) staining and morphological lipid droplet accumulation.

### Preparation of PC

To ensure solubility and bioavailability consistent with clinical pharmacological formulations, phosphatidylcholine (Lipoid S-100; Lipoid GmbH, Ludwigshafen, Germany) was prepared as a conjugate with bovine serum albumin (BSA; Sigma‒Aldrich). A PC concentration of 25 mg/mL was selected to approximate the localized pharmacological environment encountered during clinical injection lipolysis rather than systemic circulating levels. Because PC is a phospholipid with low aqueous solubility, direct addition to the medium would result in precipitation. Therefore, we utilized a method involving 0.8% fatty acid-free BSA as a carrier. Briefly, PC was dissolved in ethanol, and this solution was mixed with serum-free medium supplemented with BSA under constant vortexing. The mixture was then sonicated to form a stable, homogenous dispersion of PC vesicles/micelles. The final solution was assessed optically to confirm the absence of macroscopic aggregates or precipitation. The final concentration of ethanol in the cell culture medium was strictly maintained below 0.1% (*v/v*) in all the experimental groups to avoid any vehicle-associated cytotoxicity or interference with lipid metabolism by solvent effects.

### Drug treatment and cell viability assay

To assess cytotoxicity, fully differentiated 3T3-L1 adipocytes were seeded (0.01 × 10^6^/well) in 96-well plates. Cells were pretreated with the selective TLR4 signaling inhibitor TAK-242 (1 or 10 nM; Sigma‒Aldrich) for 4 h prior to PC exposure. This pretreatment phase allows the inhibitor to bind to the intracellular domain of TLR4 and effectively block downstream signaling. The cells were subsequently treated with 25 mg/mL PC for 24 h. Cell viability was quantitatively assessed using a water-soluble tetrazolium (WST) assay kit (DoGenBio). The WST reagent is reduced by dehydrogenases in viable cells to form a colored formazan dye. The absorbance was measured at 450 nm using a microplate reader (Bio-Rad), and the cell viability is expressed as a percentage of that of the vehicle-treated control group. The absorbance values were verified to remain within the linear range.

### Lipolysis and lipid accumulation assays

Lipolytic activity was quantified by measuring the release of glycerol, the final product of triglyceride hydrolysis, into the culture medium. The supernatants were collected after treatment, and the glycerol concentration was determined using a colorimetric glycerol assay kit (Abcam) according to the manufacturer’s instructions. The absorbance was read at 570 nm, and the values were normalized to the total protein content. All the experimental conditions were matched with those of the cytotoxicity test.

To visualize and quantify the intracellular lipid content, the cells were washed with phosphate-buffered saline (PBS) and fixed with 10% formalin for 1 h. After fixation, the cells were stained with 0.3% ORO solution (Sigma‒Aldrich) in 60% isopropanol for 30 min at room temperature. Stained cells were washed with distilled water to remove excess dye and photographed under a light microscope. For quantitative analysis, the retained Oil Red O dye was extracted with 100% isopropanol, and the absorbance was measured at 520 nm.

### Western blotting

For protein analysis, fully differentiated 3T3-L1 adipocytes cultured in 6-well plates were harvested and lysed in radioimmunoprecipitation assay (RIPA) buffer (Thermo Scientific) supplemented with a protease and phosphatase inhibitor cocktail. The lysates were subsequently centrifuged at 12,000 rpm for 20 min at 4 °C, after which the supernatants were collected. The protein concentration was determined using a BCA protein assay kit (Pierce). Equal amounts of protein (30 μg) were separated by sodium dodecyl sulfate‒polyacrylamide gel electrophoresis (SDS‒PAGE) on 10%–12% gels and transferred to nitrocellulose membranes. The membranes were blocked with 5% skim milk in Tris-buffered saline with Tween-20 (TBST) for 1 h and then probed with primary antibodies against TLR4, phosphorylated NF-κB p65 (Ser536), total NF-κB p65, TNF-α, cleaved caspase-3, and β-actin (Cell Signaling Technology; 1:1000 dilution) overnight at 4 °C. After washed with TBST, the membranes were incubated with horseradish peroxidase (HRP)-conjugated secondary antibodies for 1 h at room temperature. The protein bands were visualized using an enhanced chemiluminescence (ECL) detection system and quantified by densitometry using ImageJ software.

### Annexin V/DAPI staining for apoptosis detection

To further distinguish between programmed cell death (apoptosis) and non-specific necrosis, we performed Annexin V-FITC and DAPI (4′,6-diamidino-2-phenylindole) dual staining. Fully differentiated 3T3-L1 adipocytes were treated with PC (25 mg/mL) for 24 h in the presence or absence of TAK-242 (1 or 10 nM). After treatment, the cells were washed with cold PBS and incubated with Annexin V-FITC binding buffer containing DAPI for 15 min at room temperature in the dark, according to the manufacturer’s instructions (DoGenBio, Seoul, Korea). The stained cells were immediately visualized using a fluorescence microscope (Nikon, Tokyo, Japan). To provide a quantitative assessment, the number of Annexin V-positive apoptotic cells was counted across five randomly selected fields per unit area for each experimental group.

### Statistical analysis

The data are presented as the mean ± SEM. Comparisons between groups were performed using one-way ANOVA followed by Tukey’s *post hoc* test for multiple comparisons using GraphPad Prism software. A p value <0.05 was considered to indicate statistical significance.

## Results

### TLR4 inhibition attenuates PC-induced adipocytotoxicity and lipolysis in a dose-dependent manner

**FIGURE 1:**
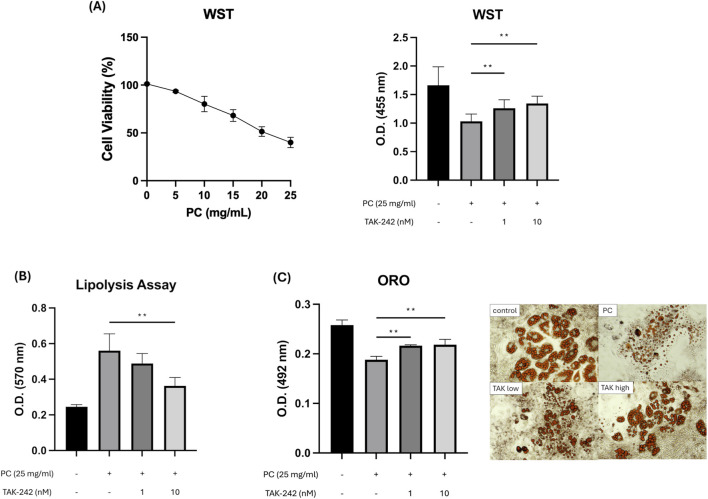
Effects of PC and TLR4 inhibition on adipocyte viability, lipolysis, and lipid accumulation. **(A)** The viability of differentiated 3T3-L1 adipocytes was assessed via a WST assay. Initially, a dose-response analysis was performed by treating cells with various concentrations of PC (0–25 mg/mL) to establish the cytotoxic profile (left panel). Subsequently, cell viability was evaluated following treatment with PC (25 mg/mL) in the presence or absence of the TLR4 inhibitor TAK-242 (1 or 10 nM) (right panel). PC treatment significantly reduced cell viability in a concentration-dependent manner, which was reversed by TAK-242 in a dose-dependent manner, indicating specific receptor-mediated cytotoxicity (n = 16). **(B)** Glycerol release, a direct indicator of lipolytic activity, was measured in the culture supernatant (absorbance at 570 nm). PC-induced glycerol release was significantly suppressed by TAK-242 pretreatment, confirming the presence of TLR4-dependent lipolysis (n = 16). **(C)** Intracellular lipid accumulation was quantified using Oil Red O (ORO) staining (absorbance at 492 nm). PC treatment resulted in a substantial reduction in lipid content, which was dose-dependently restored by TLR4 inhibition (n = 8). The data are presented as the mean ± SEM. *p < 0.05, **p < 0.01 vs. the indicated groups.

To elucidate the role of TLR4 in PC-mediated adipocyte remodeling, we first examined the effects of the specific TLR4 inhibitor TAK-242 on cell viability and lipid metabolism. Treatment of differentiated 3T3-L1 adipocytes with PC at concentrations ranging from 0 to 25 mg/mL resulted in a significant and concentration-dependent reduction in cell viability, as assessed by the WST assay. Specifically, the viability decreased to approximately 40% at 25 mg/mL of PC. Notably, pretreatment with TAK-242 (1 or 10 nM) dose-dependently mitigated this cytotoxic effect, significantly restoring the O.D. values compared to the PC-only treated group (Figure 1A). We next assessed lipolytic activity by measuring the release of glycerol, a direct indicator of triglyceride hydrolysis. PC treatment markedly elevated extracellular glycerol levels, confirming its potent lipolytic capacity (Figure 1B). Importantly, this PC-induced glycerol release was significantly suppressed by TLR4 inhibition. Consistent with these biochemical findings, Oil Red O staining revealed that PC treatment substantially decreased the number of intracellular lipid droplets (Figure 1C). This delipidation was partially reversed by TAK-242, indicating that TLR4 signaling is essential for PC-driven lipid catabolism and adipocyte loss.

### PC triggers apoptosis and activates the TLR4-NF-κB-TNF-α signaling axis

**FIGURE 2:**
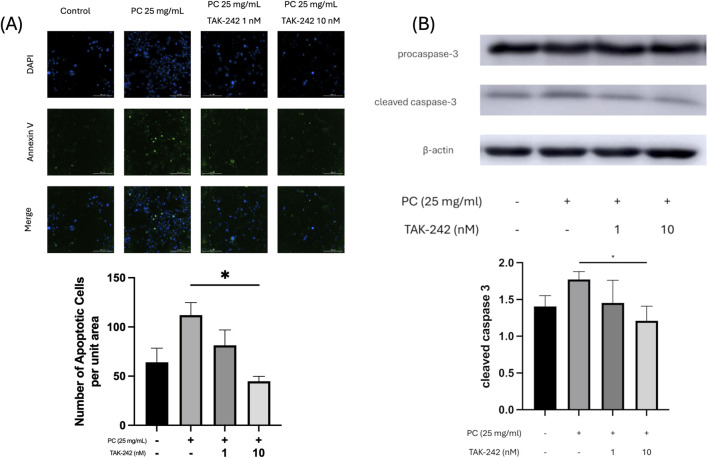
PC-induced adipocyte apoptosis is mediated via TLR4 signaling. Representative images and quantitative analysis showing the pro-apoptotic effects of PC on 3T3-L1 adipocytes (n = 3). **(A)** Annexin V (green) and DAPI (blue) fluorescence staining (top) and the quantitative analysis of the number of apoptotic cells per unit area (bottom). PC (25 mg/mL) treatment significantly increased the apoptotic cell population, an effect that was dose-dependently and significantly reversed by the TLR4 inhibitor TAK-242 (1 or 10 nM). **(B)** Representative Western blot bands for procaspase-3 and cleaved caspase-3 (top) and the corresponding densitometric analysis of cleaved caspase-3 (bottom). PC-induced activation of the executioner caspase-3 was effectively suppressed by TLR4 inhibition, confirming that PC triggers receptor-mediated programmed cell death. The data are presented as the mean ± SEM. *p < 0.05 vs. the PC-treated group.

To elucidate the molecular mechanism downstream of receptor activation, we analyzed the expression of key inflammatory and apoptotic proteins. To confirm the induction of programmed cell death, we performed Annexin V/DAPI dual staining. Fluorescence microscopy revealed a marked increase in Annexin V-positive (apoptotic) cells following PC treatment (25 mg/mL) compared to the control group. Quantitative analysis showed that the number of apoptotic cells per unit area significantly increased, an effect that was effectively and dose-dependently reversed by TAK-242 pretreatment (10 nM) (Figure 2A). Furthermore, Western blot analysis showed that the levels of cleaved caspase-3, the executioner of apoptosis, were significantly upregulated by PC but markedly reduced by TAK-242 (Figure 2B).

**FIGURE 3:**
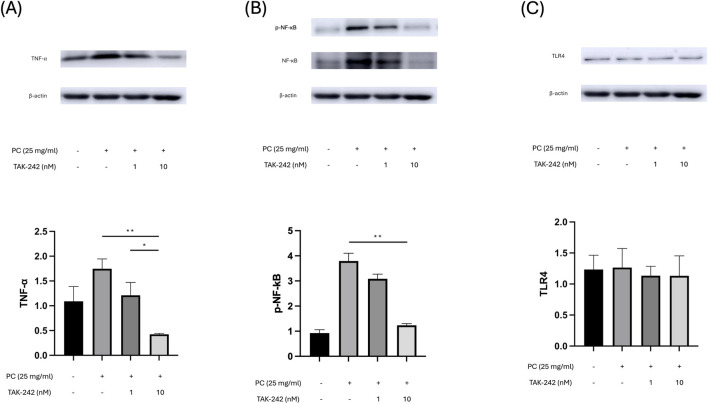
PC functionally activates the TLR4-NF-κB-TNF-α signaling axis. Representative Western blot images and densitometric analysis showing the activation of downstream inflammatory signaling in differentiated 3T3-L1 adipocytes (n = 3). **(A)** Expression levels of the pro-inflammatory cytokine TNF-α. PC treatment markedly upregulated TNF-α expression, which was significantly attenuated by TAK-242 in a dose-dependent manner. **(B)** Functional activation of the NF-κB pathway assessed by the phosphorylation of NF-κB p65 (p-NF-κB). PC treatment triggered a robust increase in p-NF-κB levels, providing definitive proof of signaling activation; this effect was effectively blocked by TAK-242. **(C)** Total protein expression of TLR4. Despite the robust activation of downstream markers, total TLR4 abundance remained unchanged across all experimental groups, suggesting that PC-mediated signaling involves functional activation of the receptor rather than changes in its protein stability. β-actin was used as a loading control. The data are presented as the mean ± SEM. *p 0.05, **p < 0.01 vs. the PC-treated group.

To provide definitive proof of functional signaling activation, we analyzed the phosphorylation of NF-κB. PC treatment triggered a robust increase in p-NF-κB levels, indicating the initiation of pro-inflammatory signaling. This activation was significantly attenuated by TAK-242 in a dose-dependent manner, confirming that PC triggers inflammation specifically via the TLR4-NF-κB axis (Figure 3B). Consistent with NF-κB activation, the expression of the pro-inflammatory cytokine TNF-α was significantly increased after PC treatment but was suppressed by TAK-242 (Figure 3A). In contrast, total TLR4 protein expression remained largely unchanged across all experimental groups (Figure 3C), suggesting that PC activates the functionality of the TLR4 cascade without altering the total abundance of the receptor itself.

## Discussion

The present study identifies PC as a functional activator of the TLR4 signaling pathway in adipocytes, providing a novel mechanistic explanation for its role in injection lipolysis. We demonstrated that treatment with PC at a pharmacological concentration (25 mg/mL) significantly reduced cell viability, enhanced glycerol release, and decreased lipid accumulation in differentiated 3T3-L1 adipocytes. Crucially, these catabolic effects were dependent on TLR4 signaling, as evidenced by their reversal with the specific inhibitor TAK-242. Our dose-response experiments (0–25 mg/mL) further validated this receptor-mediated specificity, as the concentration-dependent reduction in viability was significantly rescued by TAK-242, distinguishing it from non-specific detergent effects. These findings fundamentally shift the understanding of PC from a passive solubilizing vehicle to an active pharmacological agent that exploits innate immune pathways to drive adipose tissue remodeling.

The concept of “sterile inflammation”—an inflammatory response triggered by endogenous signals rather than pathogens—is central to understanding the mechanism of PC. Our hypothesis posits that the supraphysiological concentrations of extracellular PCs utilized in mesotherapy function as pharmacological DAMPs, recapitulating the massive release of endogenous lipids typically associated with acute cellular rupture (Gong et al., 2020; Palsson-McDermott and O'Neill, 2013). This mechanism is supported by the structural homology between phospholipid acyl chains and lipid A, the bioactive moiety of LPS (Maeshima and Fernandez, 2013; Park et al., 2009). We propose that these lipid species dock within the hydrophobic pocket of MD-2, thereby inducing TLR4 dimerization and triggering a targeted catabolic signaling cascade. While dietary lipids and free fatty acids have been implicated in chronic low-grade inflammation (meta-inflammation) associated with obesity (Shi et al., 2006; Lancaster et al., 2018), our study shows that the acute, high-dose administration of PC coopts this same pathway to achieve a therapeutic reduction in fat mass.

A particularly intriguing finding of our study was that total TLR4 protein levels remained unchanged despite robust downstream signaling. Classically, strong TLR4 activation by LPS leads to receptor endocytosis and lysosomal degradation, a phenomenon known as “endotoxin tolerance,” which is designed to prevent excessive inflammation (Zanoni et al., 2011). The absence of this downregulation in our study suggests a distinct kinetic profile for PC-mediated activation. It is possible that PC or its metabolite, as a lipid-based agonist, interacts with the receptor in a manner that triggers the MyD88-dependent pathway (leading to NF-κB activation) without efficiently engaging the CD14-dependent endocytic machinery required for receptor internalization and TRIF signaling (Kagan et al., 2008). This “biased agonism” or sustained surface expression would allow for continuous signaling and a prolonged lipolytic effect, which is clinically desirable in injection lipolysis. Alternatively, the rate of receptor-recycling from endosomes might perfectly balance the rate of internalization under these specific conditions (Husebye et al., 2006).

The downstream consequences of this TLR4 activation—specifically the upregulation of NF-κB and TNF-α—provide the functional machinery for lipolysis.

To provide definitive proof of functional signaling activation, we analyzed the phosphorylation of NF-κB (p-NF-κB). The robust increase in p-NF-κB levels following PC treatment, and its subsequent dose-dependent suppression by TAK-242, confirms the functional activation of the signaling cascade moving beyond mere protein abundance. TNF-α is a well-established lipolytic cytokine that acts through multiple mechanisms: it downregulates perilipin expression, thereby exposing lipid droplets to hydrolysis; it increases the expression and phosphorylation of HSL and ATGL; and it suppresses the expression of insulin receptors to prevent the re-esterification of fatty acids (Sethi and Hotamisligil, 1999; Langin and Arner, 2006). Our data confirms this axis, as TAK-242 not only blocked NF-κB and TNF-α induction but also rescued lipid accumulation. However, because excessive TNF-α signaling can contribute to fibrosis and adipose dysfunction, fine-tuning of TLR4 activation or partial modulation within the therapeutic window could optimize cosmetic outcomes while minimizing adverse effects. Furthermore, the robust activation of cleaved caspase-3 indicates that the terminal fate of these adipocytes is apoptosis. This was further confirmed by Annexin V/DAPI dual staining, which provided direct visual and quantitative evidence of apoptotic cell populations that were dose-dependently reduced by TLR4 inhibition. This predominant activation of apoptotic signaling pathways under the observed conditions is a distinct advantage over the necrotic mode of action by detergents such as DCs (Schuller-Petrovic et al., 2008; Klein et al., 2009). Apoptosis allows for the orderly removal of cell debris by phagocytes without the massive release of intracellular enzymes and inflammatory mediators seen in necrosis, potentially leading to less fibrosis and a smoother cosmetic outcome (Rose and Morgan, 2005).

Comparisons with previous studies highlight the importance of formulation components. While Rotunda et al. emphasized the necrotic effect of deoxycholate (Rotunda et al., 2004), our findings align with more recent work by Jung et al. and Kim et al., which demonstrated specific apoptotic effects of PC (Jung et al., 2019; Kim et al., 2017). However, our study advances the field by identifying the specific receptor (TLR4) responsible for this effect. These findings have significant clinical implications. If the therapeutic efficacy of injection lipolysis relies on TLR4-mediated apoptosis, then the variability in patient response could be linked to polymorphisms in the TLR4 gene or the baseline inflammatory state of the patient’s adipose tissue (Davis et al., 2008). Moreover, our results suggest that excessive inflammation—a common side effect of lipolysis injections—could be managed by fine-tuning the PC dose or potentially co-administering mild anti-inflammatory agents that do not completely abolish the TLR4 signal but prevent a local “cytokine storm”.

While our results demonstrate that TLR4 is the essential mediator of PC-induced lipolysis and apoptosis, further studies are needed to definitively establish direct agonism. Although the results for TAK-242 confirm that TLR4 is the primary gateway for cascade activation, whether PC itself, or a downstream metabolite, acts as the main ligand remains to be determined. Additional investigations involving MD-2 binding assays, TLR4 reporter assays, or CRISPR-Cas9-mediated TLR4 knockdown with LPS as a positive control will be instrumental in distinguishing direct receptor activation from potential membrane perturbations or the secondary release of endogenous DAMPs. Although we have now provided dose-response data and confirmed NF-κB phosphorylation and Annexin V positivity to address the previous methodological gaps, we acknowledge that while a homogeneous dispersion of PCs was visually confirmed, the precise micelle or vesicle size was not formally quantified using methods such as dynamic light scattering (DLS). We acknowledge this as a limitation, as micelle size could influence the reproducibility of subsequent *in vitro* cellular responses. Future studies should also explore the secondary necrosis at much later stages beyond 24 h. Finally, the quantification of PPARγ or adiponectin expression or triglyceride levels would have further validated the 3T3-L1 differentiation levels. Future research should also investigate whether PC activates other PRRs, such as TLR2, which has also been implicated in lipid sensing (Lancaster et al., 2018).

**FIGURE 4:**
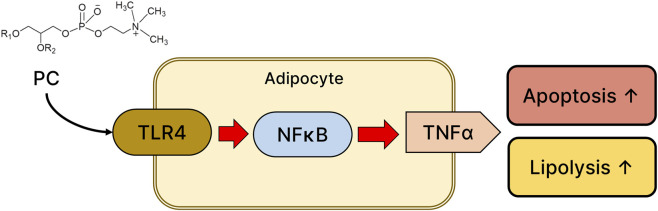
Schematic diagram of the proposed signaling pathway for PC-induced lipolysis and apoptosis in adipocytes. Extracellular phosphatidylcholine (PPC) acts as a noncanonical agonist of Toll-like receptor 4 (TLR4) on the adipocyte membrane. Upon activation, TLR4 initiates the downstream inflammatory signaling cascade, leading to the translocation of NF-κB and the subsequent upregulation of TNF-α. This signaling axis ultimately increases both lipolysis and apoptosis in adipocytes.

In conclusion, this study bridges the gap between lipid biochemistry and innate immunity in aesthetic medicine. We provide compelling evidence that extracellular PCs recreate a DAMP-abundant environment, triggering the TLR4-p-NF-κB-TNF-α axis to induce adipocyte-specific lipolysis and apoptosis (Figure 4). These findings support the use of PC as an active ingredient in lipolytic formulations and suggest that the modulation of TLR4 signaling represents a novel target for optimizing nonsurgical fat reduction therapies.

## Data Availability

The raw data supporting the conclusions of this article will be made available by the authors, without undue reservation.
